# Interventional Treatment of Pulmonary Valve Stenosis: A Single Center Experience

**DOI:** 10.3889/oamjms.2015.089

**Published:** 2015-08-25

**Authors:** Shpend Idrizi, Ivan Milev, Planinka Zafirovska, Goce Tosheski, Zan Zimbakov, Vilma Ampova-Sokolov, Tanja Angjuseva, Zan Mitrev

**Affiliations:** *Special Hospital for Surgical Diseases “Filip Vtori”, Skopje, Republic of Macedonia*

**Keywords:** percutaneous pulmonary valvuloplasty, pulmonary stenosis, interventional treatment, congenital heart disease, pulmonary regurgitation

## Abstract

**BACKGROUND:**

Percutaneous pulmonary valvuloplasty is well established treatment of choice in pulmonary valve stenosis.

**AIM:**

The aim of our study was to present our experience with the interventional technique, its immediate and mid-term effectiveness as well as its complication rate.

**MATERIAL AND METHODS:**

The study included 43 patients, where 33 (74%) of them were children between the age of 1 month and 15 years.

**RESULTS:**

The procedure was successful in 38 patients or 90%. Mean peak to peak transvalvular gradient was reduced from 91.2 mmHg (55-150 mmHg) to 39.1 mmHg (20-80 mmHg). Follow- up of patients was between 2 and 13 years and included echocardiographic evaluation of pulmonary valve gradient, right heart dimensions and function as well as assessment of pulmonary regurgitation. We experienced one major complication pericardial effusion in a 5 months old child that required pericardiocenthesis. Six patients (13.9%) required a second intervention. During the follow up period there was significant improvement of right heart function and echocardiography parameters. Mild pulmonary regurgitation was noted in 24 (55%) patients, and four (9%) patients developed moderate regurgitation, without affecting the function of the right ventricle.

**CONCLUSIONS:**

Percutaneous pulmonary valvuloplasty is an effective procedure in treatment of pulmonary stenosis with good short and mid-term results.

## Introduction

Pulmonary valve stenosis (PS) is a heart valve disorder in which outflow of blood from the right ventricle of the heart is obstructed at the level of the pulmonic valve. Although most commonly diagnosed and treated in the pediatric population, individuals with more severe forms of PS are surviving into adulthood and require ongoing assessment and cardiovascular care. The prevalence of valvular pulmonary stenosis has been reported at 0.6 to 0.8 cases per 1000 live births. When associated with other congenital heart disorder, it occurs in approximately 50% of all born with some kind of congenital heart disease [1]. PS can be due to isolated valvular (90%), subvalvular, or peripheral (supravalvular) obstruction, or it may be found in association with congenital heart disorders of some other kind [2].

### Valvular pulmonic stenosis

Isolated valvular PS comprises approximately 10% of all congenital heart disease. Typically, the valve commissures are partially fused and the 3 leaflets are thin and pliant, resulting in a conical or dome-shaped structure with a narrowed central orifice. Poststenotic pulmonary artery dilatation may occur owing to “jet-effect” hemodynamics which is a good predictor for successful outcome of interventional treatment. With severe PS and decreased right ventricular chamber compliance, cyanosis can occur from right-to-left shunting if a concomitant patent foramen ovale, atrial septal defect, or ventricular septal defect is present.

### Subvalvular pulmonic stenosis

Subvalvular PS occurs as a narrowing of the infundibular or subinfundibular region, often with a normal pulmonic valve. This condition is present in individuals with tetralogy of Fallot and can also be associatedwith a ventricularseptaldefect (VSD).

### Peripheral pulmonary stenosis

Peripheral pulmonary stenosis (PPS) can cause obstruction at the level of the main pulmonary artery, at its bifurcation, or at the more distal branches. PPS may occur at a single level, but multiple sites of obstruction are more common. PPS may be associated with other congenital heart anomalies such asvalvular PS, atrialseptaldefect (ASD), VSD, orpatentductusarteriosus (PDA); 20% of the patients with tetralogy of Fallot have associated PPS.

Functional or physiologic PPS is a common cause of a systolic murmur in infants. It occurs in both premature and full-term infants; with time, the pulmonary artery grows, and the murmur usually disappears within a few months.

Most children and adults with mild-to-moderately severe pulmonic stenosis (PS) are asymptomatic. The ones with severe PS may experience exertional dyspnea and fatigue. In extremely rare cases, patients present with exertional angina, syncope, or sudden death. Peripheral edema and other typical symptoms occur with right heart failure. Cyanosis is present in those with significant right-to-left shunt via a patent foramen ovale, atrial septal defect, or ventricular septal defect [3].

Up until 1982 this condition was treated surgically, the same year the technique of percutaneous balloon pulmonary valvuloplasty(BVP) was introduced by Kan et al. and since then it has become the established method of treatment of moderate and severe pulmonary stenosis in most cardiac centers in developed countries [4]. Its minimal invasiveness and avoidance of sternotomy are some of the advantages over open surgical treatment. Potential disadvantages of this treatment are pulmonary valve re-stenosis, rupture of the pulmonary artery walls and post interventional high grade valve insufficiency. The successful use of the procedure in adults has been reported by several investigators [5-19]. There are two major differences between balloon pulmonic valvuloplasty in adults and in children. When done in adults it does not seem as necessary as it is in children to select a balloon size substantially larger than the annulus of the pulmonic valve [20]. Second, the improvement in adults after the initial balloon dilation is usually maintained over a long term; whereas in children, there is a 19 % incidence of restenosis [21], particularly in newborns, which leads to possible redilatation of the valve.

The aim of our study was to present the rate of success and complications in percutaneous pulmonary valvuloplasty done at our clinic during a period of eleven years, both in children and in adults.

## Methods

From November 2003 to November 2014, forty three consecutive patients with moderate to severe PS, were considered for BVP. An inclusion criterion for BVP was the grade of the peak systolic pressure gradient across the pulmonic valve measured with Doppler echocardiography. We treated patients whose peak systolic gradient across the pulmonic valve was 50 mmHg or greater, with a normal cardiac index irrespective of symptoms. Thirty three children (1-15 years old), 1 adolescent and 9 adults with PS were identified at our institution. The clinical characteristics of patients were reviewed. Physical examination, chest X-ray, electro-cardiography and transthoracic echocardiography (TTE) were performed before the procedure.

**Figure 1:**
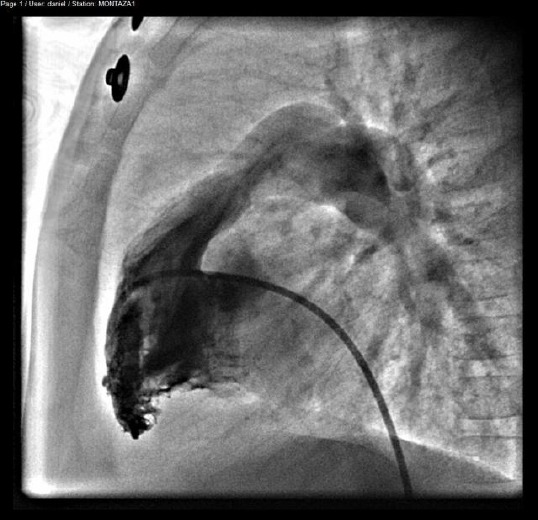
*Right ventriculography*.

The upcoming technique of balloon dilatation of the pulmonary valve has been described thoroughly [22, 23]. The intervention was done under general anesthesia with endotracheal intubation for children and local anesthesia for adults. Seldinger technique was used to insert 4-6 French pigtail catheters through the right or left femoral venous sheath along v. cava inferior into the right atrium, than in the right ventricle where right ventriculography was performed in left lateral views (Figure 1).

The lateral view of the right ventricular angiogram was used to measure the diameter of the orifice and annulus of the pulmonary valve from hinge to hinge point. The maximal diameter of the Tyshak II Balloon was purposely selected to be 1-2 mm larger than the one of the pulmonary valve annulus. This decision was made thanks to the reports of the usefulness of larger balloons [24-26] an attempt was made to achieve a balloon/annulus ratio between 1.2 and 1.4 and in the last years we tend to decrease the ratio to 1.1-1.2 due to novel studies recommending lower ratio [27]. A multipurpose catheter was then introduced into the femoral venous sheath, advanced across the pulmonary valve with the tip positioned in one of the distal pulmonary artery branches. After the exchange wire was stabilized, balloon catheter was pulled back up to the point where the balloon’s middle portion was positioned across the pulmonary valve.

**Figure 2:**
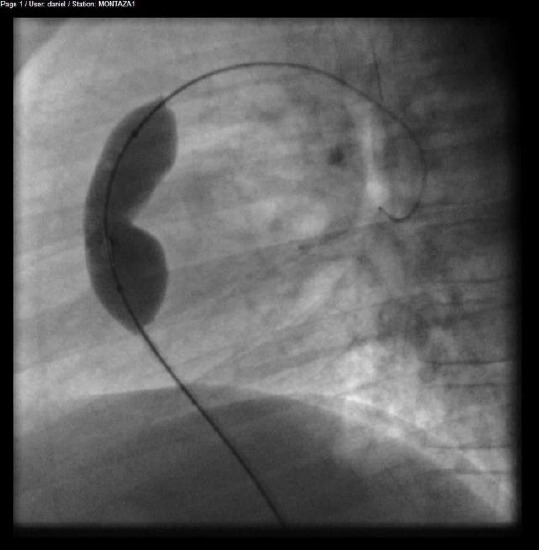
*Balloon inflation through the pulmonary valve*.

At that exact moment the balloon was fully inflated within a few seconds and then quickly deflated (Figure 2).

Right after the process of dilatation was performed, the multipurpose or MP catheter was pulled from the pulmonary artery into the right ventricle to measure the transvalvular pressure gradient. The balloon was reinflated to a larger diameter if the gradient had not substantially decreased. Finally, a pigtail catheter was inserted over the guide wire into the right side of the heart for additional right ventricular angiography. After the intervention, all adult patients were transferred to general wards and administered aspirin 3 mg/kg per day for 6 months. Control TTE was performed at one week and routinely assessed at one month after the procedure and once a year thereafter.

## Results

Percutaneous pulmonary valvuloplasty was performed on 43 patients (age 1-66 years; 65% female) with congenital pulmonary stenosis (Table 1). Nine of the pediatric patients were associated with another congenital heart defect: atrial septal defect (ASD) in three, VSD in 5 and tetralogy Fallot associated with complete atrioventricular canal defect – CAVC and sy. Down in one patient. Thirty three (84%) of our patients had valvular stenosis, one patient had subvalvular, two had supravalvular and the rest combined. Twelve patients (28%) had symptoms of fatigue. Dyspnea and cyanosis were present in 6 (14%). Other common clinical presentations were chest pain and palpitations.

**Table 1:** Demographic and characterization data of patients

Patients	43
Mean age	52.5
Age groups	
Newborns	1 (2.31%)
Babies	8 (19%)
Children	24 (55.81%)
Adolescents	1 (2.3%)
Adults	9 (21%)
Gender	
Male	15 (35%)
Female	28 (65%)
Symptomatology	
Asymptomatic	24 (58%)
Symptomatic(dyspnea.fatigue)	12 (28%)
Symptomatic with cyanosis	6 (14%)
Transpulmonary valve pressure gradient	
50-70 mmHg	7 (16%)
70-100 mmHg	26(60%)
>100 mmHg	10 (24%)
Type of stenosis	
Valvular	36 (83%)
Subvalvular	1 (2.3%)
Supravalvular	2(4.7%)
Combined	4(9.3%)

Seven patients had moderate pulmonary stenosis with peak systolic gradient across the pulmonary valve of 50-70 mmHg, twenty six had severe pulmonary stenosis (70-100 mmHg) and ten patients were treated for pulmonary stenosis above 100 mmHg.

**Figure 3:**
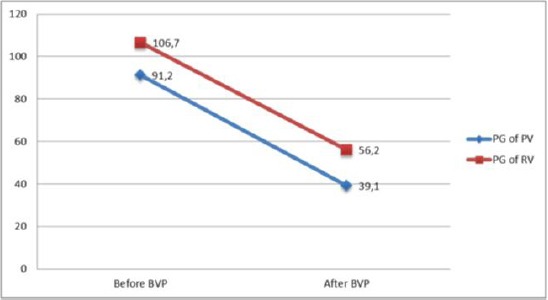
*Decrease of mean transvalvular and right ventricular pressure gradient right after the intervention(PG of RV- pressure gradient in right ventricle; PG of PV- pressure gradient of pulmonary valve; BVP- balloon valvuloplasty)*.

Pre interventional and post interventional data are shown in Figure 3.

The mean right ventricle systolic pressure before intervention was 106.7 mm/Hg and decreased to 56.2 mm/Hg after intervention. The mean peak to peak systolic pressure across pulmonary artery was 91.2 mm/Hg and 39.1 mm/Hg right after intervention.

**Figure 4:**
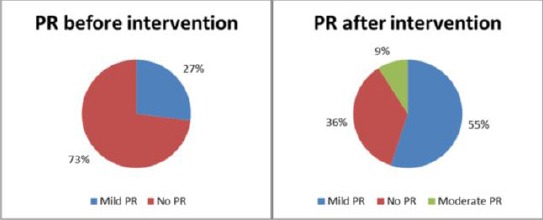
*Pulmonary regurgitation before and after intervention*.

In the patients presenting with cyanosis post-intervention their saturation improved gradually and cyanosis decreased. Changes in the right ventricle in a matter of hypertrophy were noted in 27 patients and right ventricle dilatation in 5. Regression in right ventricular dimensions was observed in all patients. In 88% of patients right heart dimension and function completely normalized at follow-up. Hypertrophy of right ventricular free wall remained in 5 patients, all of them belonging to the adult group. Mild pulmonary regurgitation was noted in 24 (55%), and 4 (9%) patients experienced moderate pulmonary regurgitation after the intervention. During our follow up period there was no incidence of severe pulmonary regurgitation (Figure 4).

We experienced one major complication - pericardial effusion in a 5 month old child that required pericardiocenthesis. Total of 6 patients (14%) required a second intervention, four after 2 years, and two after period of 3 years, due to restenosis of the pulmonary valve.

**Figure 5:**
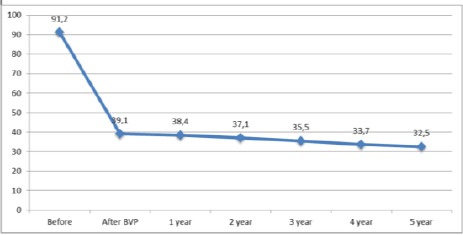
*Mean residual gradient of pulmonary artery valve at follow-up*.

Routine follow up was performed in most of the patients for a 5 year period after the intervention. The mean residual gradient in the final examination has dropped to 32.5 mmHg (Figure 5).

As stated previously re-intervention was undertaken in 5 patients. The re-intervention was unsuccessful for only one adult female patient whose transvalvular gradient remained 55 mmHg.

## Discussion

This study demonstrates our experience with the percutaneous pulmonary valvuloplasty, its immediate and mid-term relief of stenosis of the pulmonary valve in most of the patients with moderate to severe pulmonary stenosis. The procedure not only improved the hemodynamic but also the clinical status of the patients with severe form of pulmonary stenosis. Being a nonsurgical procedure is its biggest advantage and is associated with less psychological trauma. Lower incidence in morbidity and mortality as well as shorter hospital days along with its cost effectiveness makes this technique as the treatment of choice for children and adults with valvular pulmonary stenosis.

In our study, 90% of the patients had the transvalvular gradient decreased under 50mmHg with the procedure. The other patients required second intervention after 2-3 years. The second intervention was unsuccessful in one girl whose transvalvular gradient remained 55mmHg. All of patients that underwent second intervention had dysplastic pulmonary valve.

Although rare complications during or immediately after this procedure are noted in several studies. Mortality rate and major complications have been found to be around 0.24% and 0.35% respectively in a multi-center study [28]. Most common symptoms are hypotension and bradycardia that tend to resolve quickly after balloon deflation. We experienced transient arrhythmias in patients during intervention with five (12%) of our patients.

Another frequent association with PS is infundibular obstruction. This condition can usually be seen in older patients that are with higher degree of valvar obstruction, but it also tends to improve with time. Beta blockers have been used for significant infundibular obstruction > 50 mmHg after theintervention [29, 30]. Infundibular obstruction was present in 35% of our patients and was treated with beta blockers in the next 3-6 months after the procedure. At follow-up the infundibular gradients either diminished or disappeared. The infundibular gradients appeared to be more frequent with increasing age and severity of pulmonary valvar obstruction. Other complications reported in the literature are transient or permanent complete heart block, significant blood loss, seizures, cardiac arrest, cerebrovascular accidents, balloon rupture, rupture of tricuspid valve papillary muscle, and pulmonary artery tear. During the period represented in our study none of the complications above were experienced.

As in the most catheterization based intervention, there is potential for femoral vein occlusion after pulmonary valvuloplasty. It is predominantly observed in 7-19% and more often in young infants [31].

Pulmonary valve regurgitation is reported in 41-88% of cases undergoing pulmonary balloon valvuloplasty[32;33;34]. In our study the incidence of pulmonary regurgitation was 38% and the regurgitation was mild, with only one patient (0.3%) developing moderate regurgitation. During the follow- up period none of our patients developed severe pulmonary regurgitation.

In conclusion, balloon valvuloplasty is the treatment of choice in the management of moderate to severe pulmonary stenosis with excellent short- and mid-term results. Mortality and major complications are very rare. Pulmonary regurgitation is a complication with insignificant impact on right heart function at short and mid-term follow up. However, long term follow-up studies are needed, mostly for evaluation of pulmonary regurgitation.
